# Supplementary material for: Interim recruitment prediction for multi-center clinical trials
Source: Biostatistics. 2020 Sep 25;23(2):485–506. doi: 10.1093/biostatistics/kxaa036 (PMC9007446; doi:10.1093/biostatistics/kxaa036)
Supplement: kxaa036_Supplementary_Data [file kxaa036_supplementary_data.pdf]

Biostatistics (2020), **0**, 0, *pp.* 1–15
doi:10.1093/biostatistics/output

Supplementary material for Interim recruitment prediction for multi-centre clinical trials

SZYMON URBAS*

STOR-i CDT, Lancaster University, Lancaster, United Kingdom

s.urbas@lancaster.ac.uk

CHRIS SHERLOCK

Department of Mathematics and Statistics, Lancaster University, Lancaster, United Kingdom

PAUL METCALFE

AstraZeneca, Cambridge, United Kingdom

This file contains the technical appendix for "Interim recruitment prediction for multi-centre clinical trials". The algorithm for the non-parametric bootstrap test of Section 3 of the main article is outlined in Appendix A. Appendix B provides full parametric forms of the integrated intensity curve-shapes described in Section 4; it also discusses a potential identifiability problem. Appendices C and D outline the details of maximum likelihood and Bayesian inference on the model parameters. Appendix E provides the density of the prior described in Section 5.1. The time-to-completion Monte Carlo sampling algorithm is outlined in Appendix F. Appendix G provides additional details and figures for the simulation study in Section 6 and data analysis in Section 7. Appendix H describes an implementation of a centre-initiation delay model into the prediction framework.

*To whom correspondence should be addressed.

© The Author 2020. Published by Oxford University Press. All rights reserved. For permissions, please e-mail: journals.permissions@oup.com

APPENDIX

A. Non-parametric bootrapped test

Input: Series of counts $\{N_c(t)\}_{t=1}^{\tau_c}$, $c = 1, \ldots, C$; number of bootstrapped samples B.

Output: Probability of observed difference in means under H_0.

- Calculate observed difference $\Delta = \sum_{c=1}^{C} \left(\sum_{t=1}^{\tau_c/2} N_c(t) - \sum_{t=\tau_c/2+1}^{\tau_c} N_c(t) \right)$
- For $b \leftarrow 1$ to B do
  - For $c \leftarrow 1$ to C do: Resample $\{N_c^{(b)}(t)\}_{t=1}^{\tau_c}$ with replacement
  - Calculate difference $\Delta^{(b)} = \sum_{c=1}^{C} \left(\sum_{t=1}^{\tau_c/2} N_c^{(b)}(t) - \sum_{t=\tau_c/2+1}^{\tau_c} N_c^{(b)}(t) \right)$
- Calculate approximate p-value: $\hat{p} = \frac{1}{B} \sum_{b=1}^{B} \mathbb{I}_{\{\Delta \geqslant \Delta^{(b)}\}}$

B. Curve-shape

The integrated, normalised parametric intensities are:

$$
\begin{aligned}
G_0(t) &= t, \\
G_1(t;\theta) &= \frac{\log(1+\theta t)}{\log(1+\theta\tau)}\tau, \\
G_\kappa(t;\theta) &= \frac{(1+\theta t/\kappa)^{1-\kappa} - 1}{(1+\theta\tau/\kappa)^{1-\kappa} - 1}\tau, \quad \kappa \notin \{0, 1, \infty\}, \\
G_\infty(t;\theta) &= \frac{1-\exp\{-\theta t\}}{1-\exp\{-\theta\tau\}}\tau.
\end{aligned}
$$

In two instances, the flexible-tail form can give rise to identifiability problems:

<u>$t, \tau >> \kappa/\theta$ and $\kappa < 1$</u>

$$
G_\kappa(t;\theta) = \frac{(1+\theta t/\kappa)^{1-\kappa} - 1}{(1+\theta\tau/\kappa)^{1-\kappa} - 1}\tau \approx \frac{(\theta t/\kappa)^{1-\kappa} - 1}{(\theta\tau/\kappa)^{1-\kappa} - 1}\tau \approx \left(\frac{t}{\tau}\right)^{1-\kappa}\tau,
$$

which does not depend on θ.

<u>$t, \tau >> \kappa/\theta$ and $\kappa >> 1$</u>

$$
\begin{aligned}
G_\kappa(t;\theta) &= \frac{(1+\theta t/\kappa)^{1-\kappa}-1}{(1+\theta\tau/\kappa)^{1-\kappa}-1}\tau \\
&\approx \frac{(1+\theta t/\kappa)\exp\{-\theta t\}-1}{(1+\theta\tau/\kappa)\exp\{-\theta\tau\}-1}\tau \\
&\approx \frac{\exp\{-\theta t\}-1}{\exp\{-\theta\tau\}-1}\tau,
\end{aligned}
$$

which does not depend on κ.

C. Maximum likelihood inference

In the frequentist setting, we aim to find estimators which maximise the likelihood surface (4.2) in the main paper. This is equivalent to maximising the log-likelihood surface (up to a constant)

$$
\begin{aligned}
\ell(\alpha,\phi,\theta|\mathbf{n},\boldsymbol{\tau}) = C\left(\alpha\log\frac{\alpha}{\phi}-\log\Gamma(\alpha)\right) - \sum_{c=1}^{C}\Bigg\{\left(\alpha+n_c^{(\cdot)}\right)\log\left(G(\tau_c;\theta)+\frac{\alpha}{\phi}\right) \\
-\log\Gamma\left(\alpha+n_c^{(\cdot)}\right) - \sum_{t=1}^{\tau_c} n_c^{(t)}\log(G(t;\theta)-G(t-1;\theta))\Bigg\}.
\end{aligned}
$$

The log-likelihood function can be optimised using a range of methods, for example, the Nelder-Mead (Nelder and Mead, 1965) method used in R. The inverse of the negative Hessian at the mode can then be used as the covariance matrix for the asymptotic normal distribution of the MLEs.

The α and ϕ parameters are asymptotically orthogonal for a homogeneous Poisson-gamma model (Huzurbazar, 1950). A time contraction argument can be used to extend the result to the inhomogeneous case. As discussed in Section 4 of the main paper and visible from (4.3), in the special case where $\tau_c \equiv \tau \ \forall c$, θ is orthogonal to both α and ϕ. When carrying out maximum likelihood inference, different model selection criteria such as AIC (Akaike, 1973) and BIC (Schwarz, 1978) can be used. Alternatively, one could employ frequentist model averaging methods (see Hjort and Claeskens (2003), for instance).

The score function and the observed and expected information are provided in the Supple-

mentary Material. The only pair of parameters which are not asymptotically orthogonal when centres have not been open for the same length of time are ϕ and θ.

Score and observed and expected information

Here we provide the score function and the observed and expected information, for frequentist inference.

The score function is the gradient of the log-likelihood of the model,

$$\nabla\ell(\alpha,\phi,\theta|\mathbf{n},\boldsymbol{\tau}) = \\ = \begin{bmatrix} C\left(1+\log\frac{\alpha}{\phi}-\psi(\alpha)\right)-\sum_{c=1}^{C}\left(\frac{\alpha+n_c^{(\cdot)}}{\alpha+\phi G(\tau_c;\theta)}+\log\left(G(\tau_c;\theta)+\frac{\alpha}{\phi}\right)-\psi\left(\alpha+n_c^{(\cdot)}\right)\right)\tau \\ -C\alpha/\phi+\sum_{c=1}^{C}\frac{\alpha\left(\alpha+n_c^{(\cdot)}\right)}{\phi(\alpha+\phi G(\tau_c;\theta))}\tau \\ -\sum_{c=1}^{C}\left[\partial_\theta G(\tau_c;\theta)\left(\frac{\alpha+n_c^{(\cdot)}}{G(\tau_c;\theta)+\frac{\alpha}{\phi}}\right)-\sum_{t=1}^{\tau_c}n_c^{(t)}\left(\frac{\partial_\theta G(t;\theta)-\partial_\theta G(t-1;\theta)}{G(t;\theta)-G(t-1;\theta)}\right)\right]\end{bmatrix}.$$

The observed information matrix is made up of the negative Hessian elements

$$\begin{aligned}
-\partial^2_{\alpha\alpha}\ell(\alpha,\phi,\theta|\mathbf{n},\boldsymbol{\tau}) =& C\left(\psi'(\alpha)-\frac{1}{\alpha}\right)+\sum_{n=1}^{C}\left\{\frac{\phi G(\tau_c;\theta)-n_c^{(\cdot)}+1}{\alpha+\phi G(\tau_c;\theta)}-\psi'\left(\alpha+n_c^{(\cdot)}\right)\right\},\\
-\partial^2_{\phi\phi}\ell(\alpha,\phi,\theta|\mathbf{n},\boldsymbol{\tau}) =& -C\alpha/\phi^2+\sum_{c=1}^{C}\frac{\alpha\left(\alpha+2\phi G(\tau_c;\theta)\right)\left(\alpha+n_c^{(\cdot)}\right)}{\phi^2(\alpha+\phi G(\tau_c;\theta))^2},\\
-\partial^2_{\theta\theta}\ell(\alpha,\phi,\theta|\mathbf{n},\boldsymbol{\tau}) =& \sum_{c=1}^{C}\left[\frac{\left(\alpha+n_c^{(\cdot)}\right)\left\{\partial^2_{\theta\theta}G(\tau_c;\theta)(G(\tau_c;\theta)+\alpha/\phi)-(\partial_\theta G(\tau_c;\theta))^2\right\}}{(G(\tau_c;\theta)+\alpha/\phi)^2}\right.\\
&\left.-\sum_{t=1}^{\tau_c}n_c^{(t)}\frac{H_t\partial^2_{\theta\theta}H_t-(\partial_\theta H_t)^2}{(H_t)^2}\right],\\
-\partial^2_{\alpha\phi}\ell(\alpha,\phi,\theta|\mathbf{n},\boldsymbol{\tau}) =& \frac{1}{\phi}\left\{C-\sum_{c=1}^{C}\frac{\alpha^2+2\alpha\phi G(\tau_c;\theta)+\phi G(\tau_c;\theta)n_c^{(\cdot)}}{\left(\alpha+\phi G(\tau_c;\theta)\right)^2}\right\},\\
-\partial^2_{\alpha\theta}\ell(\alpha,\phi,\theta|\mathbf{n},\boldsymbol{\tau}) =& \sum_{c=1}^{C}\partial_\theta G(\tau_c;\theta)\frac{G(\tau_c;\theta)-n_c^{(\cdot)}/\phi}{\{G(\tau_c;\theta)-\alpha/\phi\}^2},\\
-\partial^2_{\phi\theta}\ell(\alpha,\phi,\theta|\mathbf{n},\boldsymbol{\tau}) =& -\alpha\sum_{c=1}^{C}\partial_\theta G(\tau_c;\theta)\frac{\alpha+n_c^{(\cdot)}}{\{\alpha+\phi G(\tau_c;\theta)\}^2},
\end{aligned}$$

where $\psi(x)=\Gamma'(x)/\Gamma(x)$ and $H_t=G(t;\theta)-G(t-1;\theta)$ to simplify the notation. Noting that

$E\left[N_c^{(\cdot)}\right] = \phi G(\tau_c;\theta)$, we obtain the entries of the Fisher information matrix,

$$
\begin{aligned}
E[-\partial^2_{\alpha\alpha}\ell(\alpha,\phi,\theta|\mathbf{N},\boldsymbol{\tau})] =& C\left(\psi'(\alpha) - \frac{1}{\alpha}\right) + \sum_{n=1}^{C}\left[\frac{1}{\alpha+\phi G(\tau_c;\theta)} - E\Big\{\psi'\left(\alpha + n_c^{(\cdot)}\right)\Big\}\right],\\
E[-\partial^2_{\phi\phi}\ell(\alpha,\phi,\theta|\mathbf{N},\boldsymbol{\tau})] =& \frac{\alpha}{\phi}\sum_{c=1}^{C}\frac{G(\tau_c;\theta)}{\alpha+\phi G(\tau_c;\theta)},\\
E[-\partial^2_{\theta\theta}\ell(\alpha,\phi,\theta|\mathbf{N},\boldsymbol{\tau})] =& \sum_{c=1}^{C}\left[\frac{\phi\left\{\partial^2_{\theta\theta}G(\tau_c;\theta)(G(\tau_c;\theta)+\alpha/\phi) - (\partial_\theta G(\tau_c;\theta))^2\right\}}{\phi G(\tau_c;\theta)+\alpha}\right.\\
&\left. - \sum_{t=1}^{\tau_c} n_c^{(t)}\partial^2_{\theta\theta}H_t - \frac{(\partial_\theta H_t)^2}{H_t}\right],\\
E[-\partial^2_{\alpha\phi}\ell(\alpha,\phi,\theta|\mathbf{N},\boldsymbol{\tau})] =& 0,\\
E[-\partial^2_{\alpha\theta}\ell(\alpha,\phi,\theta|\mathbf{N},\boldsymbol{\tau})] =& 0,\\
E[-\partial^2_{\phi\theta}\ell(\alpha,\phi,\theta|\mathbf{N},\boldsymbol{\tau})] =& -\alpha\sum_{c=1}^{C}\frac{\partial_\theta G(\tau_c;\theta)}{\alpha+\phi G(\tau_c;\theta)}.
\end{aligned}
$$

D. Bayesian inference

For a general model with data y, parameter vector $\psi \in \Omega$ and likelihood $f(y|\psi)$, we assign a prior density or mass function to ψ, $\pi_0(\psi)$. Inference is based on the posterior distribution, obtained by the Bayes's rule,

$$\pi(\psi|y) = \frac{f(y|\psi)\pi_0(\psi)}{\int_\Omega f(y|\psi)\pi_0(\psi)\,\mathrm{d}\psi}, \quad \psi \in \Omega.$$

Often times, the marginal likelihood of the data $p(y) = \int_\Omega f(y|\psi)\pi_0(\psi)\,\mathrm{d}\psi$ is not tractable and so Monte Carlo sampling methods need to be employed to obtain samples from the posterior. Strictly, the marginal likelihood, $p(y)$ is $p(y|M)$ the probability of the data given the choice of model, encapsulated in f. Consider, now, a range of models $M_1,\ldots,M_K$ with associated prior probabilities $\pi_0(M_k)$, $k = 1,\ldots,K$. Using Bayes's rule, we obtain the posterior model probabilities, up to a proportionality constant,

$$\pi(M_k|y) \propto p(y|M_k)\pi_0(M_k), \quad k = 1,\ldots,K.$$

Importance sampling

Multiplying the priors and the likelihood we obtain the posterior distribution for the parameters up to a proportionality constant. Since the dimension of the parameter space is not large, we can sample from the posterior by the means of importance sampling.

For any function of interest $h(\psi)$,

$$\begin{aligned}\mathbb{E}[h(\psi)] &= \int_\Omega h(\psi)\pi_p(\psi|y)\ \mathrm{d}\psi = \frac{\int_\Omega h(\psi)\omega(\psi)q(\psi)\ \mathrm{d}\psi}{\int_\Omega f(y|\psi)\pi_0(\psi)\ \mathrm{d}\psi} \\ &\approx \frac{\sum_{b=1}^B h\left(\psi^{(b)}\right)\omega\left(\psi^{(b)}\right)}{\sum_{b=1}^B \omega\left(\psi^{(b)}\right)},\end{aligned}$$

where $\psi^{(b)}$, $b = 1, \ldots, B$ are samples from a proposal distribution q with unnormalised weights

$$\omega(\psi) = \frac{f(y|\psi)\pi_0(\psi)}{q(\psi)}.$$

The marginal likelihood may be approximated by

$$\hat{p}(y) = \frac{1}{B}\sum_{b=1}^B \omega\left(\psi^{(b)}\right).$$

This is an unbiased estimate which can be used for model selection or model averaging.

The efficiency of the sampling procedure depends on the choice of proposal distribution q and the may be measured using the effective sample size (ESS),

$$\mathrm{ESS} = \frac{\left(\sum_{b=1}^B \omega\left(\psi^{(b)}\right)\right)^2}{\sum_{b=1}^B \omega\left(\psi^{(b)}\right)^2}.$$

If the proposal distribution closely resembles the true posterior, then all the weights will be roughly the same resulting in the ESS being close to M. On the other extreme, if the proposal badly captures the posterior and one sample's weight dominates the others, then ESS will be close to one.

If $\psi^{(b)}$ are resampled with replacement with probabilities proportional to the weights, then the resulting sample, say $\{\psi_*^{(b)}\}_{b=1}^B$, will have the distribution approximating π. The new sample is used when sampling from the predictive distribution to marginalise over the parameter posterior.

E. Curve-shape prior

The flexible form (4.4) in Section 4 of the main paper, leads to the following prior density for $\tilde{\theta}$,

$$\pi_0\left(\tilde{\theta}|\kappa, a, b\right) = \begin{cases} t_0 \exp\left\{\tilde{\theta} - t_0 \exp\{\tilde{\theta}\}\right\} f_{\mathcal{B}}\left(\exp\left\{-t_0 \exp\{\tilde{\theta}\}\right\}; a, b\right), & \kappa = \infty \\ t_0 \exp\{\tilde{\theta}\}\left(1 + t_0 \exp\{\tilde{\theta}\}/\kappa\right)^{-\kappa-1} f_{\mathcal{B}}\left(\left(1 + t_0 \exp\{\tilde{\theta}\}/\kappa\right)^{-\kappa}; a, b\right), & \kappa \in (0, \infty) \end{cases},$$

where $f_{\mathcal{B}}(\cdot; a, b)$ is a density of a beta variate with shape parameters a and b.

F. Sampling time to completion via model averaging

In Lan *and others* (2018), the time to recruit the required number of patients is sampled by repeatedly simulating the whole system until the condition is satisfied, which is inefficient because each iteration involves a (random) large number of expensive simulations. Additionally, it only provides an approximate distribution due to the discretisation in the time domain; the discretisation of recruitment to monthly increments might also affect the precision of any predictions. To sample the time to completion exactly, we use the integrated intensity function of the whole trial $\Lambda(t)$. If T is the time to the mth arrival of an inhomogeneous Poisson process with integrated intensity $\Lambda(t)$ then (Devroye, 1986)

$$\Lambda(T) \sim \text{Gamma}(m, 1).$$

Given (α, β, θ), we can sample rates the λ_c^o for all the centres and construct one realisation of the integrated intensity Λ for the whole trial. Then, to obtain a single realisation of T, we sample a Gamma$(m, 1)$ variate and use an inverse-transform of Λ on it. Unless all the centres had been open for the same length of time, the inversion procedure will involve some root-finding algorithm, such as Nelder-Mead (Nelder and Mead, 1965). As $\Lambda(t)$ in our framework is a monotonically increasing function, the non-linear equation will have a unique solution. Parameter uncertainty can be incorporated into this predictive by using a different sample from the posterior at each iteration.

Given C^+ centres with the first C already opened before the census time and the remaining $C^+ - C$ to be open, as well as known centre opening times $t_0^{(c)},\ c = 1, \ldots, C^+$, we construct the integrated intensity for modelling the recruitment since the census time τ,

$$\Lambda(t) = \sum_{c=1}^{C} \lambda_c^o \left\{ G\left(t - t_0^{(c)}; \theta\right) - G(\tau_c; \theta) \right\} + \sum_{c=C+1}^{C^+} \lambda_c^o G\left(t - t_0^{(c)}; \theta\right) \chi_{\left\{t > t_0^{(c)}\right\}}, \quad t \geqslant \tau,$$

where $\chi_{\{\cdot\}}$ is the indicator function and

$$\lambda_c^o | \alpha, \phi, \theta, \mathbf{n} \sim \begin{cases} \text{Gamma}\left(\alpha + n_c^{(\cdot)}, \alpha/\phi + G(\tau_c; \theta)\right), & c = 1, \ldots, C \\ \text{Gamma}\left(\alpha, \alpha/\phi\right), & c = C+1, \ldots, C^+ \end{cases}. \tag{F.1}$$

The algorithm below outlines the sampling procedure to obtain the distribution of the time needed to recruit the target number of patients m.

Input: Models $M_1, \ldots, M_K$ with posterior probabilities $\pi(M_1|\mathbf{n}), \ldots, \pi(M_K|\mathbf{n})$ and posterior samples from each model, number of samples from the predictive B, target number of recruitments m

Output: Distribution of the time to completion $\left\{T^{(b)}\right\}_{b=1}^{B}$

For $b \leftarrow 1$ to B do:

- Sample $M^{(b)} \sim \pi(M_k|\mathbf{n})$
- Sample $(\alpha, \phi, \theta)^{(b)} \sim \pi(\alpha, \phi, \theta | M^{(b)}, \mathbf{n})$
- Sample rates $\lambda_c^o | (\alpha, \phi, \theta)^{(b)}$ from distributions (F.1) and construct $\Lambda^{(b)}(t)$
- Sample $\tilde{T} \sim \text{Gamma}(m, 1)$ and solve $\Lambda^{(b)}(T) = \tilde{T}$ Set $T^{(b)} = T$

G. Additional details from the simulation study and data analysis

G.1 *Simulation study*

Figure 1 shows the plots of posterior samples of the model. The three parameters are close to orthogonal as discussed in Sections 4 and 5 of the paper, and this approximate independence

was also observed in the posteriors of other models. Figure 2 shows a QQ-plot of the hierarchical gamma distribution compared to the posterior means of the random effects. The approximately straight line indicates that generating rates for newly opened centres from the gamma distribution will be consistent with what has been observed thus far. Figure 3 shows a QQ-plot of the theoretical, negative binomial distribution of recruitments in the first 2 months compared the observed distribution ($t_* = 60$). The theoretical distribution used the posterior means of the parameters, and the prior random effect distribution was used. The straight line shows that the model can predict the recruitment in the first two months of a centre sufficiently well. In practice, the two diagnostics would indicate that the mixing gamma distribution is sufficient and that the model is capable of accurately predicting recruitments in the early days of a new centre.

Figures 4, 5, 6 and 7 show the diagnostic plots for models fit to simulated datasets at the census $t = 360$ with the true random-effect distribution being a mixture. For $E[\lambda_c^o] = 0.01$, the relationship is close to linear and is reflected in the reasonably accurate predictions shown in the article. The QQ-plots for $E[\lambda_c^o] = 0.03$ show stronger non-linearity and informing us of the potential misspecification, thus showing that the diagnostics can be used to validate the model.

Figures 8 and 9 show examples of recruitment predictions when the random effects have a mixture distribution and the centre opening times are "clumped" together. The clumping accentuates the effect of the misspecification; the fitted model relies on the "incorrect" prior gamma distribution when simulating rates for unopened centres.

Figures 10 and 11 show the predictions made when using data simulated from a Weibull-shape intensity with centre opening times clumped together. With repeated simulations, we found a consistent correspondence between linear QQ-plots and accurate predictions.

G.2 *Data analysis*

In the dataset examined in Section 7 of the main paper, we encountered an unexpected surge in recruitments at a global scale. Figure 12 shows the accrual along with 2 sets of forecasts, focusing on the surge at a time of around 0.7. Once this has been observed, and forward predictions are needed, one possibility is modelling this as a global surge in recruitment; that is, during the period between 0.6 and 0.75 all recruitment rates are multiplied by $\exp\{\beta\}$ for some unknown β, which would be an extra parameter to be estimated via importance sampling.

H. Stochastic centre-initiation times

The framework, as presented in the main paper, is conditioned on the set of initiation times both for clarity of presentation and because it is the methodological contribution from the paper. In practice, the exact future initiation times would be unknown; instead, the practitioners would have proposed initiation schedules, contingency plans and recruitment data up to the census time. Here we present a simulation study similar to that in Section 6 of the main paper which illustrates how a stochastic centre-initiation model can be seamlessly incorporated. The centres are not initiated exactly on schedule but, instead, there is a Weibull-distributed initiation delay for each centre. Following information provided to us from a large meta-analysis, we set the Weibull parameters such that the 5th and 95th percentiles are 10 and 322 days respectively; the median delay is 90 days. At the census, the observed day-censored delays are used for maximum-likelihood estimation of the Weibull parameters; additionally centres which were planned to initiate before the census but did not do so contribute with a censored likelihood. The estimates are then used in the Monte Carlo simulations. Figure 13 compare the predictions under three different approaches; (i) the correct Weibull distribution for delays (with parameters estimated from the data), (ii) a constant, avergae delay taken to be the sample mean of the observed delays, and (iii) an assumption of no delays. It is clear that assuming no future delays given historical

evidence of the contrary leads to poor forecasts. However, even very simple delay predictions based on the empirical average can achieve desirable forecasts. Of course, fitting the true model results in predictions which capture the truth extremely well. This illustrates that our site-level prediction method can be easily combined with site-initiation models.

References

Akaike, Hirotogu. (1973). Information theory and an extension of the maximum likelihood principle. Petrov BN, Csaki F, editors. Second International Symposium on Information Theory. Budapest (Hungary): Akademiai Kiado. pp. 267–281.

Devroye, Luc. (1986). *Non-uniform Random Variate Generation*. New York: Springer-Verlag.

Hjort, Nils Lid and Claeskens, Gerda. (2003). Frequentist model average estimators. *Journal of the American Statistical Association* **98**(464), 879–899.

Huzurbazar, Vasant Shankar. (1950). Probability distributions and orthogonal parameters. Volume 46, Mathematical Proceedings of the Cambridge Philosophical Society. Cambridge University Press. pp. 281–284.

Lan, Yu, Tang, Gong and Heitjan, Daniel F. (2018). Statistical modeling and prediction of clinical trial recruitment. *Statistics in Medicine* **1–11**.

Nelder, John A and Mead, Roger. (1965). A simplex method for function minimization. *The computer journal* **7**(4), 308–313.

Schwarz, Gideon. (1978). Estimating the dimension of a model. *The Annals of Statistics* **6**(2), 461–464.

[!p]

[*Received August 1, 2010; revised October 1, 2010; accepted for publication November 1, 2010*]

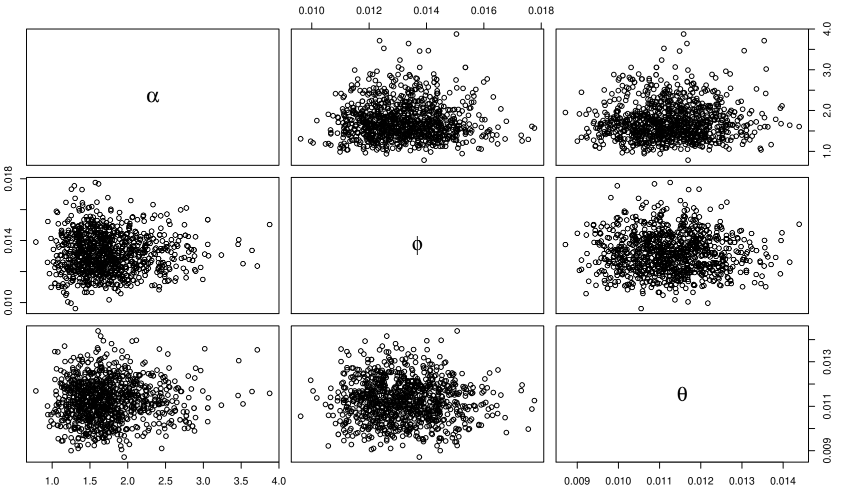

Fig. 1. Matrix scatterplot of the parameter posterior of the model with highest posterior probability.

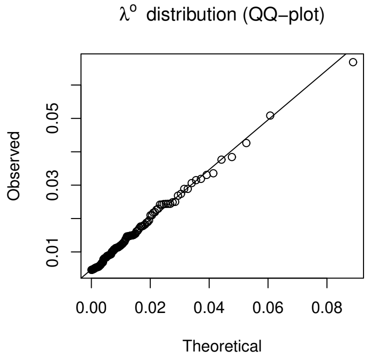

Fig. 2. Re-estimated λ_c^o expectations compared to Gamma$\left(\hat{\alpha}, \hat{\alpha}/\hat{\phi}\right)$ distribution.

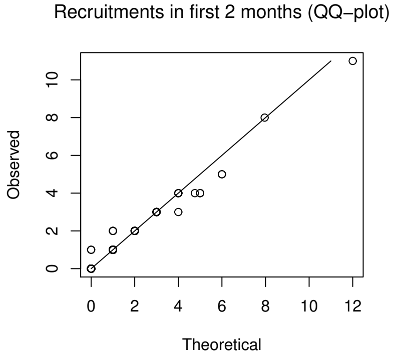

Fig. 3. Observed recruitments compared to the theoretical negative binomial distribution.

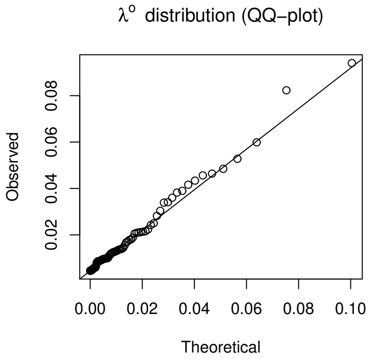

Fig. 4. Re-estimated λ_c^o expectations compared to Gamma$\left(\hat{\alpha}, \hat{\alpha}/\hat{\phi}\right)$ distribution; true random-effect distribution is a mixture with $E[\lambda_c^o] = 0.01$.

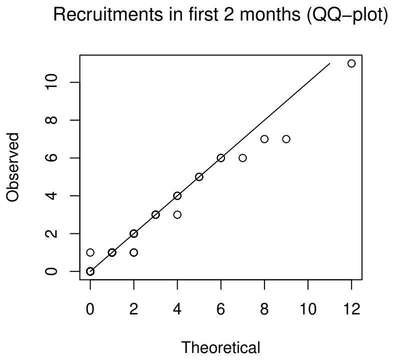

Fig. 5. Observed recruitments compared to the theoretical negative binomial distribution; true random-effect distribution is a mixture with $E[\lambda_c^o] = 0.01$

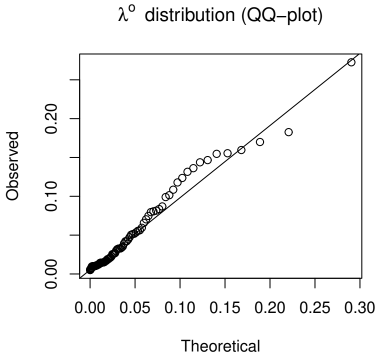

Fig. 6. Re-estimated λ_c^o expectations compared to Gamma$\left(\hat{\alpha}, \hat{\alpha}/\hat{\phi}\right)$ distribution; true random-effect distribution is a mixture with $E[\lambda_c^o] = 0.03$.

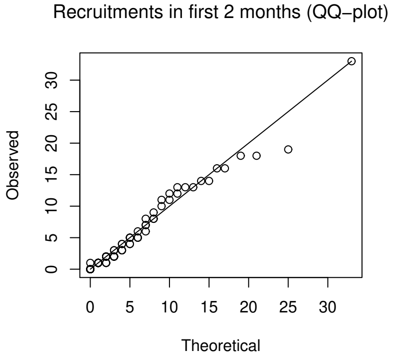

Fig. 7. Observed recruitments compared to the theoretical negative binomial distribution; true random-effect distribution is a mixture with $E[\lambda_c^o] = 0.03$

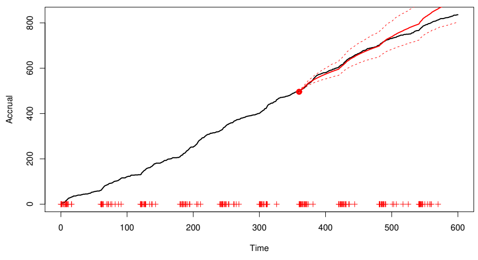

Fig. 8. Clumped openings, $E[\lambda_c^o] = 0.01$ (mixture RE distribution)

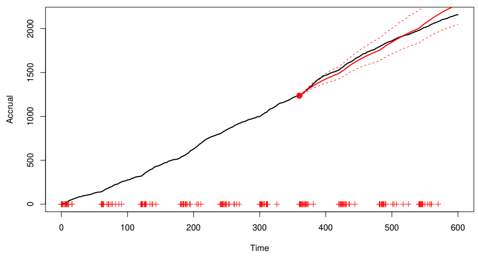

Fig. 9. Clumped openings, $E[\lambda_c^o] = 0.03$ (mixture RE distribution)

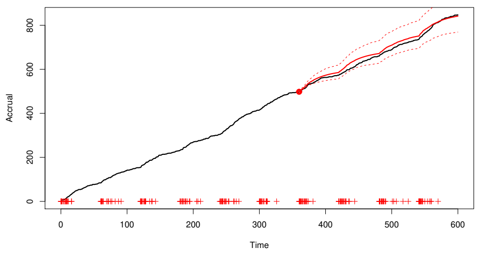

Fig. 10. Clumped openings, $E[\lambda_c^o] = 0.01$ (Weibull-shape intensity)

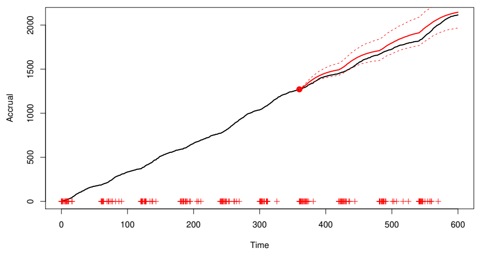

Fig. 11. Clumped openings, $E[\lambda_c^o] = 0.03$ (Weibull-shape intensity)

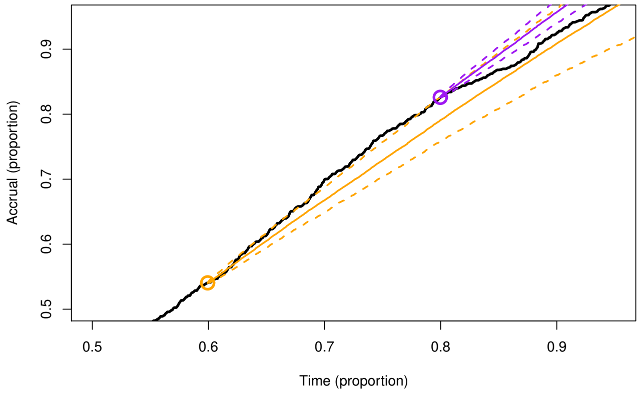

Fig. 12. Accrual predictions, zoomed-in to focus on the unexpected surge in recruitment at around the time of 0.7. Only interim forecasts from times 0.6 and 0.8 are shown.

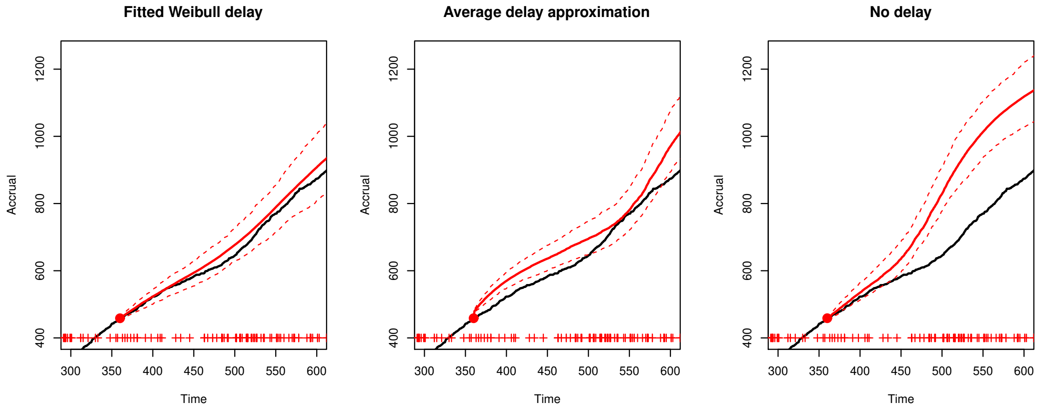

Fig. 13. Comparison of predictions for recruitment data with stochastically-delayed centre-initiation times. Three modelling approaches are considered: correct Weibull-distributed delay fitted (left); constant, historical average delay added to each initiation time (centre); and no delay considered in predictions (right).
